# Seneca Valley Virus 3C^pro^ Substrate Optimization Yields Efficient Substrates for Use in Peptide-Prodrug Therapy

**DOI:** 10.1371/journal.pone.0129103

**Published:** 2015-06-12

**Authors:** Linde A. Miles, W. Nathaniel Brennen, Charles M. Rudin, John T. Poirier

**Affiliations:** 1 Department of Pharmacology and Molecular Sciences, Johns Hopkins University, Baltimore, Maryland, United States of America; 2 Department of Oncology, Johns Hopkins University, Baltimore, Maryland, United States of America; University of North Carolina at Greensboro, UNITED STATES

## Abstract

The oncolytic picornavirus Seneca Valley Virus (SVV-001) demonstrates anti-tumor activity in models of small cell lung cancer (SCLC), but may ultimately need to be combined with cytotoxic therapies to improve responses observed in patients. Combining SVV-001 virotherapy with a peptide prodrug activated by the viral protease 3C^pro^ is a novel strategy that may increase the therapeutic potential of SVV-001. Using recombinant SVV-001 3C^pro^, we measured cleavage kinetics of predicted SVV-001 3C^pro^ substrates. An efficient substrate, L/VP4 (k_cat_/K_M_ = 1932 ± 183 M^-1^s^-1^), was further optimized by a P2’ N→P substitution yielding L/VP4.1 (k_cat_/K_M_ = 17446 ± 2203 M^-1^s^-1^). We also determined essential substrate amino acids by sequential N-terminal deletion and substitution of amino acids found in other picornavirus genera. A peptide corresponding to the L/VP4.1 substrate was selectively cleaved by SVV-001 3C^pro^ *in vitro* and was stable in human plasma. These data define an optimized peptide substrate for SVV-001 3C^pro^, with direct implications for anti-cancer therapeutic development.

## Introduction

Oncolytic viruses are replication competent viruses that selectively infect and lyse cancer cells while causing little to no harm to normal tissues [1]. Because of their inherent selectivity, the majority of oncolytic viruses are well tolerated by patients even at high doses. One such virus, Seneca Valley Virus (SVV-001), is an oncolytic picornavirus that infects tumors with neuroendocrine features, including small cell lung cancer (SCLC) and pediatric neuroendocrine tumors, with high selectivity [2]. SVV-001 has been shown to be effective in the eradication of solid tumors in multiple *in vivo* models and shows promising safety and efficacy signals in the clinic [3–6]. Initial data suggest that SVV-001 can home to the tumor and replicate specifically within tumor cells; however, neutralizing antibodies against SVV-001 develop consistently within weeks of exposure [6, 7]. Continued intratumoral viral replication may persist even after development of neutralizing antibodies, but has not been associated with long-term clinical benefit.

One strategy to harness the cancer selective activity of SVV-001 and improve its efficacy is to combine virotherapy with a peptide prodrug selectively activated at the site of infection by a virus-encoded protease, 3C^pro^. A peptide prodrug contains a peptide substrate moiety linked to a cytotoxic agent in a way that inactivates the drug. Only after selective cleavage of the peptide moiety by the target protease does the cytotoxic agent become active. Most protease activated peptide prodrugs utilize intrinsic human cellular proteases found preferentially in the target cell type versus all others [8]. In terms of novel cancer therapies, the vast majority of protease activated peptide prodrugs have targeted proteases preferentially or exclusively overexpressed in cancer cells. For instance, the prostate-specific antigen (PSA) and prostate-specific membrane antigen (PSMA) have been popular targets in prostate cancer therapeutics as their expression is relatively restricted and markedly increased in patients with prostate cancer [9–12].

**Fig 1:**
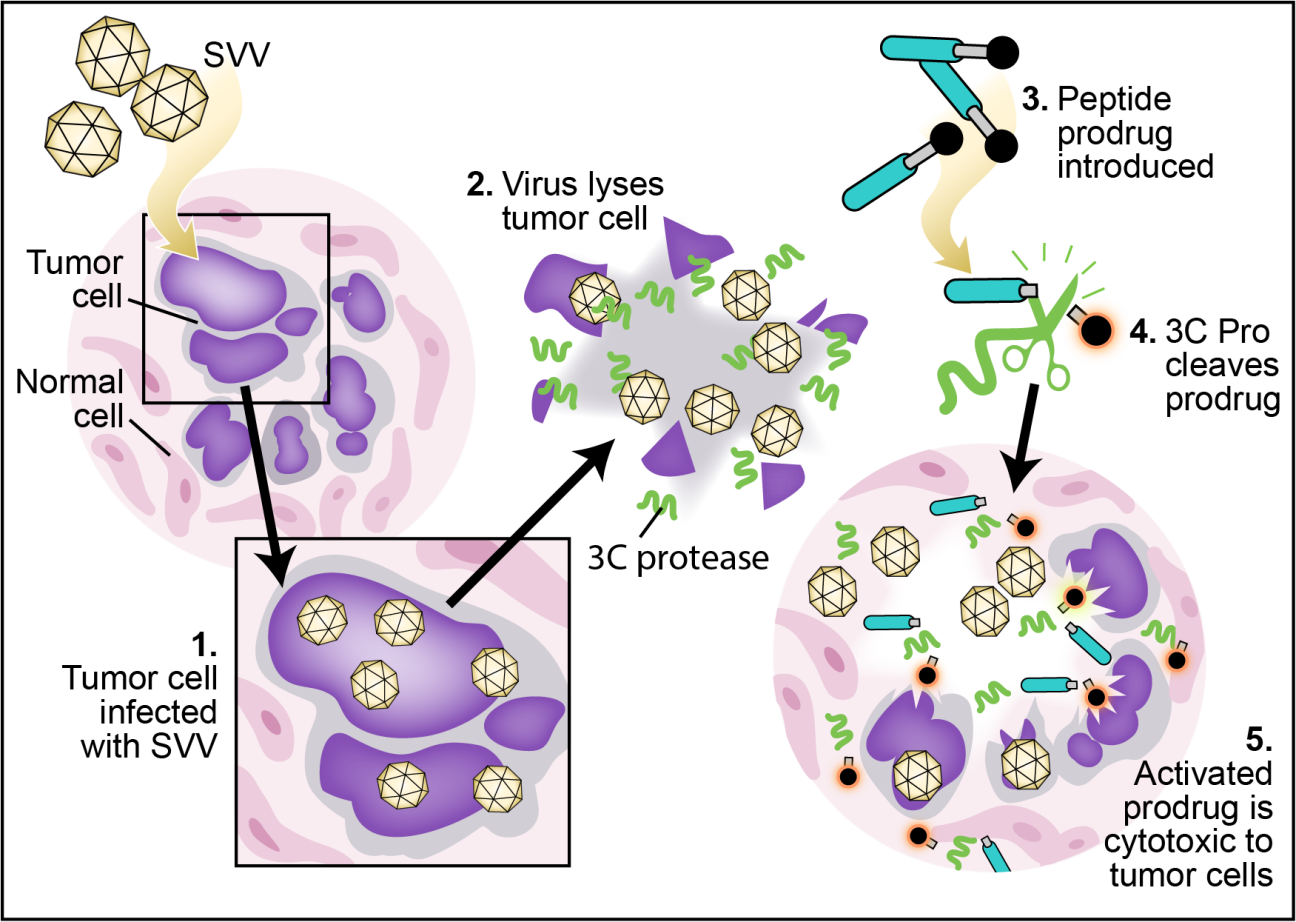
Conceptual schematic of use of an SVV 3C^pro^ activated peptide prodrug in combination with SVV virotherapy as a novel form of VDEPT. SVV infects a fraction of tumor cells (1), producing 3C^pro^ during the viral life cycle. Upon cell lysis, new SVV virions and 3C^pro^ are released into nearby tissue (2). Administered peptide prodrug would be excluded from cells by the presence of the attached peptide (3), sparing normal tissues, which are non-permissive and therefore cannot express 3C^pro^. The 3C^pro^ present at high concentration exclusively within the tumor microenvironment cleaves this peptide sequence (4), allowing the cytotoxic moiety to enter both infected and adjacent uninfected cells within the tumor, resulting in a powerful local bystander effect (5).

Alternatively, a genetically engineered virus can be used to introduce an exogenous activating enzyme into the cell of choice. This type of therapy is known as virus-directed enzyme prodrug therapy (VDEPT) [13, 14]. While not previously studied, we hypothesized that it would be possible to use a naturally occurring virally encoded protease, such as SVV-001 3C protease, for the same purpose. The SVV-001 genome is a positive sense single-stranded RNA, which is translated into a single polyprotein by the host translational machinery [2, 3, 15]. The resulting polyprotein is processed into mature proteins in part by 3C^pro^, a virus-encoded cysteine protease which contains a universally conserved His, Asp/Glu, Cys catalytic triad [16–20]. The goal of the present study was to identify optimized peptide substrates for SVV-001 3C protease and to demonstrate selective peptide hydrolysis *in vitro* as the basis for developing a novel VDEPT strategy (Fig 1). Instead of using a virus simply as a transport or targeting vector for the enzyme gene, this approach harnesses the intrinsic activity of SVV-001 3C^pro^ already existing in the wild-type SVV-001 proteome. The selectivity of the oncolytic virus for the tumor site and the existence of the active 3C^pro^ produced by the virus obviates the need for genetic engineering of the virus to carry an enzyme gene into the cells.

In this study, we quantitatively characterized the proteolytic cleavage of predicted endogenous SVV-001 3C^pro^ substrates *in vitro* using a FRET fusion protein approach. We discovered essential positions and optimal amino acid residues within one of the endogenous substrate sequences of the SVV polyprotein [20, 21]. Once we identified a substrate with high turnover efficiency, we confirmed the ability of the substrate, L/VP4.1, to be recognized and cleaved in a cellular assay by the 3C^pro^ produced during an active SVV-001 infection in the context of both a FRET fusion protein and a fluorogenic peptide. Finally, we determined the stability of the L/VP4.1 substrate in human serum for use in the development of a peptide prodrug. In summary, these studies have identified an optimized substrate to be used as the peptide in the 3C^pro^ activated peptide-prodrug as a novel VDEPT approach.

## Materials and Methods

### Reagents and Bacterial Strains

Polymerase chain reactions (PCRs) were carried out using a GeneAmp PCR System 9700 Thermocycler (Invitrogen). PCR fragments were purified using QIAquick PCR Purification Kit (Qiagen). All restriction and ligation enzymes were purchased from New England Biolabs, Inc. Competent DH10B and BL21 AI cells were purchased from Invitrogen. Plasmids were isolated and purified from bacteria using QIAquick Spin Miniprep Kit (Qiagen). Recombinant HRV 3C Protease was purchased from Sigma. Nucleotide and protein sequence alignments were performed in Geneious Pro 4.7.6. The GenBank/EMBL/DDBJ accession number for the complete genome sequence of SVV-001 is DQ641257. Genome sequences of related picornaviruses were obtained from NCBI GenBank (RefSeq. IDs NC_001366.1, NC_001479.1, NC_009448.2, NC_010810.1, NC_011349.1).

### Plasmid Construction

#### FRET Substrates

The fluorescent FRET protein pair CyPet and YPet were used to construct SVV-001 3C^pro^ substrates. The plasmid pBad33CGSYK, a gift from Dr. Patrick Daugherty, which expresses CyPet and YPet with a C-terminal 6×His tag separated by a flexible linker (SGGSGST), a non-hydrolyzable linker (NHL; GGSGGS), and a second flexible linker (GGGSGGS), was used as a template for constructing SVV-001 3C^pro^ substrates. Using forward primers 1–21 and reverse primer 22 (S1 Table) the PCR fragments were amplified to encode the corresponding substrates. Purified PCR fragments were then digested with *KpnI* and *SphI* and ligated into a similarly digested pBad33CGSYK to yield circularized plasmids containing each of the substrates flanked by a pair of flexible linkers and the CyPet and YPet proteins. Ligated plasmids were then transformed into competent DH10B cells and clones were selected on LB agar (Sigma) plates supplemented with 34 μg/ml chloramphenicol (Sigma). Substrates were verified by Sanger sequencing.

#### SVV-001 3C^pro^ Plasmid

SVV-001 3C^pro^ was cloned by fusion to Maltose Binding Protein (MBP) via a Tobacco Etch Virus (TEV) protease sequence (ENLYFQG) in a bacterial expression vector using Gateway Cloning (Invitrogen) to create a 6×His-MBP-TEV-SVV-001 3C^pro^ fusion protein plasmid [22–24]. The plasmid pNTX-09, expressing the full-length viral cDNA, was used as a template for PCR [7]. The PCR fragment was amplified using forward primer 23 and reverse primer 24, incorporating attB1 and attB2 sites for Gateway cloning. The purified PCR fragment was then used in a BP reaction (Invitrogen) with pDONR221, a destination Gateway plasmid with a kanamycin resistance gene. The recombinant plasmid was then transformed into DH10B cells and clones were selected on LB agar plates supplemented with 50 μg/ml kanamycin (Sigma). The purified plasmid containing the PCR fragment was used in a LR reaction (Invitrogen) with pDEST566 (Addgene plasmid #11517) [24]. The plasmid expresses an N-terminal 6×His MBP. The recombinant plasmid was then transformed into DH10B cells and clones were selected on LB agar plates supplemented with 100 μg/ml carbenicillin (Sigma). The purified pDEST566 plasmid containing the PCR fragment (6×His-MBP-TEV-SVV-001 3C^pro^) was then transformed into BL21 AI cells and clones were selected on LB agar plates supplemented with 100 μg/ml carbenicillin. The catalytic dead mutant 3C^pro^, SVV-001 C160A 3C^pro^, was cloned using QuikChange site-directed mutagenesis (Stratagene) using primers 25 and 26 and 6×His-MBP-TEV-SVV-001 3C^pro^ plasmid described above. The template DNA was digested by DpnI and the mutant plasmid was transformed into DH10B cells and subsequently into BL21 cells as described above. The protease sequence and the C160A mutation were confirmed using Sanger sequencing.

### Protein Induction and Purification

#### FRET Substrates

Overnight cultures of DH10B cells transformed with FRET substrate expression vectors were diluted 1:50 and grown at 37°C for 3 h in LB medium (Sigma) supplemented with 34 μg/ml chloramphenicol. Expression of FRET proteins was induced with 0.1% wt/vol L(+)-arabinose (Sigma) for 16 h at room temperature. Cells were harvested by centrifugation and soluble protein was then isolated using B-PER Protein Extraction Reagent (Pierce). Fusion proteins, which contain a C-terminal 6×His tag, were then purified using HisPur Ni-NTA resin (Pierce) and eluted in Phosphate Buffered Saline (PBS, Quality Biological), pH 7.4 with 150 mM imidazole (Sigma).

#### SVV-001 3C^pro^ Protein

Overnight cultures of BL21 AI cells transformed with pDEST566 expression vector containing the 6×His-MBP-TEV-SVV-001 3C^pro^ and 6×His-MBP-TEV-SVV-001 C160A 3C^pro^ fusion protein were diluted 1:100 and grown at 37°C until the culture reached mid-log growth phase in LB media supplemented with 100 μg/ml carbenicillin. Protein expression was induced via the addition of 1 mM isopropyl-β-D-thiogalactopyranoside (IPTG, Thermo Fisher Scientific) and 0.2% wt/vol L(+)-arabinose for 8 h at room temperature. Cells were harvested and soluble proteins were isolated as above. The fusion proteins were then purified using HisPur cobalt resin (Pierce) and eluted in PBS, pH 7.4 with 150 mM imidazole. The fractions containing the desired proteins were pooled into a 10K MWCO Slide-A-Lyzer dialysis cassette (Pierce) and dialyzed overnight at 4°C against 25 mM Tris-HCl (Thermo Fisher Scientific) pH 7.5, containing 50 mM NaCl (Thermo Fisher Scientific) and 1 mM dithiothreitol (DTT, Thermo Fisher Scientific). Dialyzed proteins were then concentrated using Amicon Ultra-15 50K MWCO centrifugal filter units (Millipore). Protein concentrations were determined using the Micro BCA Protein Assay (Pierce) and aliquots were frozen at -80°C after addition of glycerol to 10% (Sigma). Overexpression and purifications were monitored by SDS PAGE (S1 Fig). The purity of the 6×His-MBP-TEV-SVV-001 3C^pro^ preparation was estimated to be 91% using a GS-800 Calibrated Densitometer (BioRad).

### In Vitro Cleavage Assay

#### FRET Substrate Cleavage Assay and Kinetic Data Analysis

Reactions were performed in PBS, pH 7.4 and 200 nM FRET substrate in a total volume of 100 μL. Substrates were incubated with 250 nM SVV-001 3C^pro^ at 30°C for 3 h. Human rhinovirus (HRV) control substrate was incubated with recombinant HRV 3C^pro^ under the same conditions. Fluorescence emissions at 475 nm and 527 nm, corresponding to CyPet and YPet respectively, were followed after excitation at 433 nm every 180 s by a Safire fluorimeter (Tecan). All experiments were performed in triplicate. FRET ratios (YPet fluorescence/CyPet fluorescence) were calculated at each timepoint. Conversion of each substrate to cleaved substrate was calculated by dividing the change in the FRET ratio at each timepoint by the total change in the FRET ratio corresponding to complete cleavage of the substrate. Using the equation from Boulware et al., conversion data was plotted vs. time (s) and fit with the equation:

Conversion=1-

where [E] is the 3C^pro^ concentration (M) and *t* is time (s) [25]. The second order rate constant (*k*_cat_/*K*_M_) was determined by the curve fit using GraphPad Prism software. Reported values are the average *k*_cat_/*K*_M_ values of 5–7 experiments. Uncertainty is expressed by standard deviation. Error bars on data points were removed for figure clarity.

#### Fluorescently Quenched Peptide Cleavage Assay and Kinetic Data Analysis

Peptides corresponding to the substrate sequences L/VP4.1 and non-hydrolysable linker (NHL) were synthesized between a 5-carboxyfluorescein (5-FAM) fluorophore/CPC Quencher (CPQ2) quencher pair (CPC Scientific). Additional lysines were added for increased solubility giving final peptide sequences of CPQ2-IVYELQGP-K(5FAM)-KK-NH2 and CPQ2-GGSGGS-K(5FAM)-KK-NH2 for L/VP4.1 and NHL, respectively. The identity and sequence of the L/VP4.1 and NHL peptides were confirmed by CPC Scientific via LC-MS and determined to be 96.7% and 95.1% pure, respectively. The peptides were diluted in H_2_O to concentrations ranging from 7 μM to 300 nM and incubated with 50 nM purified SVV-001 3C^pro^ for 1 h at 37°C. Fluorescence emissions at 520 nm, corresponding to the liberated 5-FAM fluorophore peptide cleavage product was followed after excitation at 490 nm by a DTX 880 multimode detector (Beckman Dickinson) every 60 s. Experiments were performed in triplicate in a 96 well flat bottom black opaque plate. Standard curves of 5-FAM fluorescence vs. concentration were generated to convert the relative fluorescence units to moles of cleaved product generated during the reaction. Initial rates of hydrolysis were calculated from data during the first 3 minutes of the reaction. The initial rates of reaction from 3–4 separate experiments were then averaged and used to calculate kinetic rate constants. Standard deviation values were calculated from 3–4 separate experiments. Kinetic constants *K*_M_ and V_max_ were calculated by plotting initial rates of hydrolysis vs. substrate concentration and fitting plots with the Michaelis-Menten equation using GraphPad Prism software. Using the equation *V*_*max*_ = *k*_*cat*_ × [*E*_*t*_] and solving for *k*_cat,_, we were then able to determine the second order rate constant, *k*_cat_/*K*_M_.

### SVV-001 Cellular Assay

All SVV-001 stocks were cultured and purified as described previously and virus titers were determined by tissue culture infective dose (TCID_50_) [5]. The SCLC cell line, NCI-H446 (ATCC) was cultured in RPMI 1640 media (Quality Biological) supplemented with 10% fetal bovine serum (HyClone). The cell line NCI-H446 is routinely tested and authenticated by short tandem repeat (STR) analysis by DDC Medical and was last authenticated six months before submission.

#### FRET Substrate Cellular Assay

Cells (1.6 × 10^7^) were plated on a 150 mm cell culture dish (Corning) and allowed to recover overnight at 37°C. Each plate was inoculated with SVV-001 at the TCID_50_ and infected cells were incubated at 37°C for 8 h. After aspiration of the media, cells were mechanically lifted from the plate in phenol-red free RPMI 1640 media (Gibco) and pelleted by centrifugation. The cell pellet was then resuspended in phenol-red free RPMI 1640 media and aliquoted into the wells of a 96-well flat bottom black opaque plate (Corning). FRET substrates, L/VP4.1 or NHL, were added at a final concentration of 200 nM and incubated for 3 h at 37°C. During the substrate incubation the change in FRET was measured with a Tecan Safire fluorimeter as described above. Conversion of the L/VP4.1 substrate to cleaved substrate was calculated by dividing the change in the FRET ratio at each time point by the total change in the FRET ratio corresponding to complete cleavage of the substrate. The conversion of the NHL substrate was similarly determined by dividing the change in the FRET ratio of the NHL at each time point by the total change in the FRET ratio of the L/VP4.1 substrate corresponding to complete cleavage. Using the previously calculated second order rate constant for L/VP4.1 and the conversion equation described previously, the concentration of SVV-001 3C^pro^ was determined [25]. The reported values are averages of 7 experiments. Uncertainty is expressed by standard deviation. Error bars on data points were removed for figure clarity.

#### Fluorescently Quenched Peptide Cellular Assay

A similar cellular assay was performed as described in the previous paragraph with the exception of using the CPQ2/5-FAM L/VP4.1 and NHL fluorogenic peptides described earlier. After an 8 hour infection with SVV-001, NCI-H446 cells were removed from the cell culture dish using phenol-red free RPMI 1640 media supplemented with 0.1% ethylenediaminetetraacetic acid (EDTA, Sigma) to aid in gentle cell detachment, and pelleted via centrifugation. Pellets were resuspended in phenol-red free RPMI 1640 prior to plating in black opaque 96 well plates as above. CPQ2/5-FAM peptides, L/VP4.1 or NHL were added at a final concentration of 3 μM to plated cells and incubated for 3 hours at 37°C. Fluorescence emissions at 520 nm, corresponding to the liberated 5-FAM fluorophore peptide cleavage product was followed after excitation at 490 nm by a Synergy Neo multimode plate reader (Biotek) every 60 s. The experiment was performed in triplicate in a 96 well flat bottom black opaque plate and average fluorescence units were used to observe changes in fluorescence. Standard curves of 5-FAM fluorescence vs. concentration were generated to convert the relative fluorescence units to moles of cleaved product generated during the reaction. Initial rates of hydrolysis were calculated from data during the first 10 minutes of the reaction and averaged from 3–4 separate experiments. Uncertainty is expressed by standard deviation and shown with error bars.

### Plasma Stability Assay

Human plasma was obtained from pooled discarded clinical samples less than 6 hrs old. Human plasma was isolated from whole blood by centrifugation, pooled, and diluted 1:1 with distilled H_2_O (Gibco). The L/VP4.1 and NHL fluorogenic peptides (20 μM) were incubated with plasma for 1 h at 37°C. Fluorescence emissions were obtained as described above by a DTX 880 multimode detector (Beckman Dickinson) every 60 s. Experiments were performed in triplicate in a 96 well flat bottom black opaque plate and the average fluorescence units were used to observe changes in fluorescence. Uncertainty is expressed by standard deviation.

## Results

**Fig 2:**
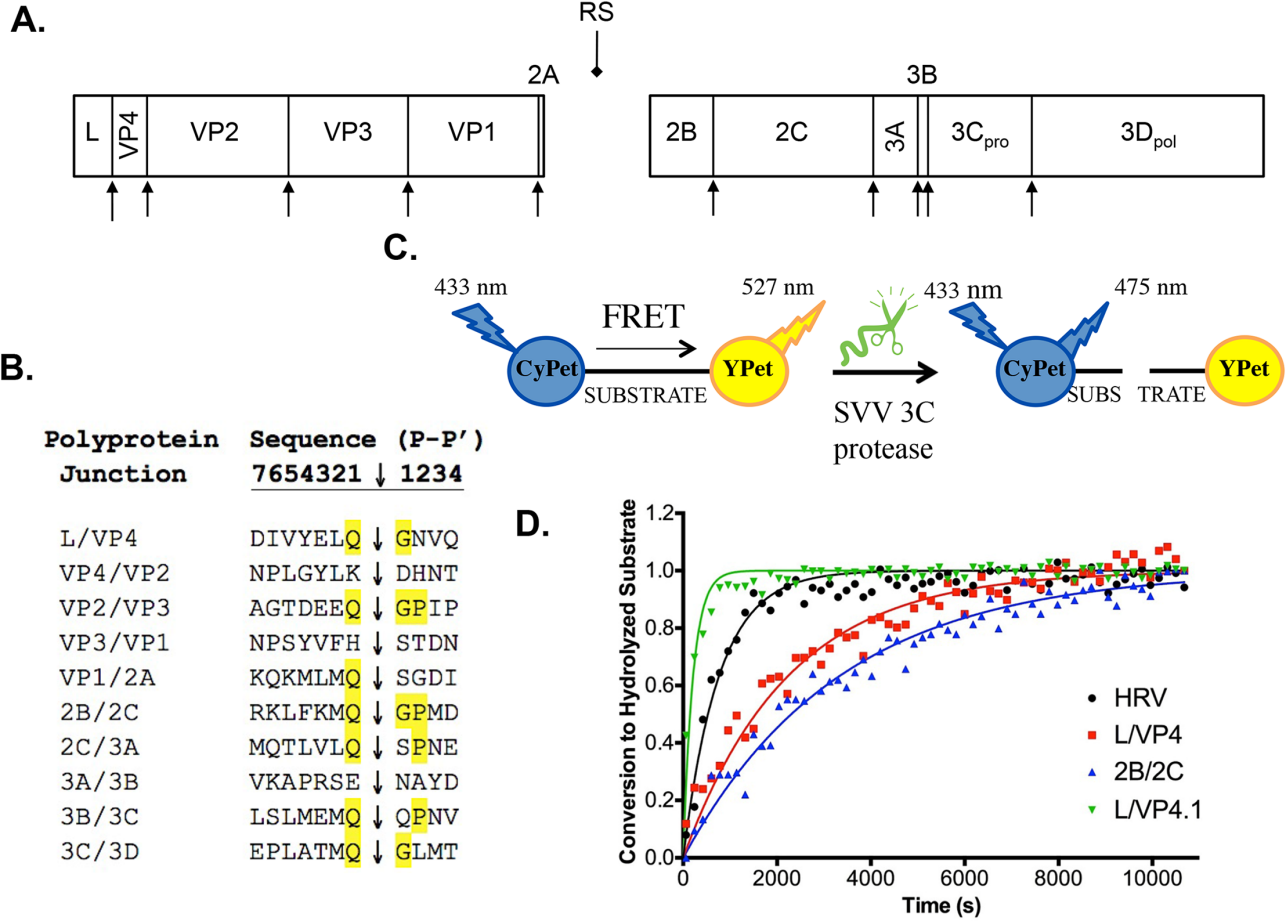
SVV-001 polyprotein and identification of efficient 3C protease substrates A. The SVV-001 polyprotein is hypothesized to include twelve mature proteins based on sequence alignment with closely related viruses. Protein regions (drawn to scale) are presented with proposed 3C^pro^ cleavage sites (arrowheads) depicted. The 2A ribosome skipping sequence (RS; diamondhead arrow) is also shown. **B.** Sequence alignment of proposed 3C^pro^ cleavage sites. Sites are named based on the two mature proteins flanking these sites. The presumed scissile bond is depicted with an arrowhead with the consensus Q↓GP cleavage sequence amino acids highlighted. **C.** Schematic of FRET substrate construction and kinetic assays. Substrates are cloned between two fluorescent proteins, CyPET and YPET. These molecules exhibit FRET when in close proximity; therefore, there will be a high level of emission at 527 nm (YPET) and lower emission at 475 nm (CyPET). As the 3C protease cleaves and releases YPET from proximity to CyPET, the amount of FRET will decrease observed as an increase in CyPET emission (475 nm) and a decrease in YPET emission (527 nm). **D.** Conversion of FRET substrates by purified HRV or SVV-001 3C^pro^ over time. Data points represent the average of three replicates at each time point. The HRV substrate was used as a positive control. L/VP4 and 2B/2C are endogenous substrates. L/VP4.1 (P2’ N→P substitution) is a further optimized version of the endogenous L/VP4 substrate. Lines of the same color correspond to curve fits from GraphPad. Error bars on data points were removed for figure clarity.

Based on sequence alignment to closely related cardioviruses, the SVV-001 polyprotein has been predicted to consist of twelve proteins after cleavage (Fig 2A). While cleavage sites for the structural proteins VP1-4 have been confirmed by N-terminal sequencing, other putative 3C^pro^ cleavage sites have been proposed based entirely on sequence homology (Fig 2B) [20]. The consensus Gln-Gly-Pro cleavage motif (Q↓GP) found for other picornaviruses is present in many predicted substrates; however, cleavage sites without this consensus sequence are also predicted [20]. As the endogenous sequences were expected to have the highest probability of being efficiently cleaved by the SVV-001 3C^pro^, our initial kinetic studies focused on the ten intrinsic substrates to identify the substrate with the highest turnover efficiency.

To identify an optimized peptide sequence for cleavage by SVV-001 3C^pro^_,_ two approaches to measure peptide cleavage were pursued. The first approach utilized FRET as a reporter for protease-catalyzed peptide cleavage. Each of the ten predicted viral cleavage sites were cloned between the FRET donor/acceptor pair, CyPet and YPet, to create FRET fusion protein substrates [21, 25, 26]. In this context, FRET is dependent on maintaining close proximity of the CyPet and YPet pairs: cleavage of the fusion proteins by purified SVV-001 3C^pro^ should result in loss of FRET (Fig 2C). The decrease in FRET was measured in real time using a fluorimeter, and second order rate constants (*k*_cat_/*K*_M_) were determined as described previously [25]. We chose an 8 residue window spanning P6-P2’ for all studies based on structural and functional studies of other picornavirus 3C proteases implicating a greater importance of non-prime side residues on substrate turnover efficiency [19, 27]. The SVV-001 3C^pro^ was cloned and purified as a recombinant fusion construct with a 6x His-tagged maltose binding protein (MBP). A non-hydrolysable linker FP substrate (NHL; GGSGGS) and an optimized human rhinovirus 3C^pro^ FP substrate (HRV; LEVLFQGP) were used as negative and positive controls, respectively, the latter being incubated with recombinant HRV 3C^pro^ [25, 27]. Interestingly, only two of the ten endogenous substrates, L/VP4 and 2B/2C, were efficiently cleaved by SVV-001 3C^pro^ *in vitro* in the context of a FP substrate. All other substrates failed to show measureable cleavage over 3 hours of incubation with the protease at 30°C. The kinetics of substrate hydrolysis or conversion were calculated (Fig 2D). Both the 2B/2C and L/VP4 FP substrates were determined to have similar *k*_cat_/*K*_M_ values at 1204 ± 146 M^-1^s^-1^ and 1932 ± 183 M^-1^s^-1^, respectively. The optimized HRV FP substrate displayed the highest turnover efficiency (*k*_cat_/*K*_M_ = 5079 ± 528 M^-1^s^-1^), which was ~3-4-fold higher than either SVV FP substrate (Table 1). Incubations of the 2C/3A, 3B/3C, and 3C/3D FP substrates with an increased protease concentration of 1 μM over three hours did demonstrate slow but nearly complete cleavage (S2 Fig). All other FP substrates showed no measurable cleavage by higher concentrations of SVV-001 3C^pro^.

**Table 1:** Summary of second order rate constants (k_cat_/K_M_) for the two endogenous substrates, controls and L/VP4 amino acid substitution/truncation mutants.

Substrate	Sequence	k_cat_/K_M_ (M^-1^s^-1^)	SD	Relative to WT
L/VP4	IVYELQGN	1932	183	---
2B/2C	KLFKMQGP	1204	146	---
HRV	LEVLFQGP	5079	528	---
NHL	GGSGGS	N/A	N/A	---
L/VP4.1	IVYELQG**P**	17446	2203	9.0
L/VP4.2	IVYE**P**QG**P**	N/A	N/A	N/A
L/VP4.3	IV**F**ELQG**P**	12160	2157	6.3
L/VP4.4	IV**M**ELQG**P**	10344	1186	5.3
L/VP4.5	VYELQG**P**	9839	696	5.1
L/VP4.6	YELQG**P**	1939	304	1.0
L/VP4.7	ELQG**P**	N/A	N/A	N/A
L/VP4.8	LQG**P**	N/A	N/A	N/A

The altered substrates are shown with the amino acid(s) substitution(s) or truncations shown in bold. Reported values are the average *k*_cat_/*K*_M_ values of 5–7 experiments. Uncertainty is expressed by standard deviation.

Surprisingly, of the two efficiently cleaved substrates, only the 2B/2C substrate contains the consensus Q↓GP motif typically found in picornavirus 3C^pro^ substrates. The L/VP4 substrate has a variant Q↓GN cleavage site, which did not appear to cause deleterious effects on the kinetics of hydrolysis. To investigate the contribution of the P2’ amino acid position, we exchanged the P2’ amino acid of 2B/2C and L/VP4 to create 2B/2C.1 (2B/2C with Q↓GN) and L/VP4.1 (L/VP4 with Q↓GP, Table 1). These new synthetic FP substrates were incubated with SVV-001 3C^pro^ and the kinetics of conversion were calculated as described above (Fig 2C and Table 1). While substituting Asn for Pro in the P2’ position of the 2B/2C substrate abolished hydrolysis, the P2’ position Pro substitution in the L/VP4 substrate increased the *k*_cat_/*K*_M_ to 17446 ± 2203 M^-1^s^-1^, an approximately 9-fold increase. We mutated the catalytic cysteine residue of SVV 3C^pro^ to an alanine (C160A 3C^pro^) to eliminate any proteolytic activity and confirmed the change in FRET of the FP substrates was entirely due to the activity of the 3C^pro^ (S3 Fig). To assess the specificity of L/VP4.1, we tested the capacity for the proteases from SVV, HRV, and Coxsackie A virus (CAV) to cleave one another’s native substrates. Interestingly, while cross-selectivity was observed between HRV and CAV, we observed no turnover of L/VP4.1 by any non-native 3C protease (Figure A and B in S4 Fig). Additionally, we tested the ability of the SVV 3C^pro^ to cleave the alternative cleavage sequence motif Q↓SP by cloning a P1’ GN→SP substituted form of L/VP4 and confirmed it had an increased turnover efficiency over L/VP4, but was inferior to L/VP4.1 (L/VP4.9; S5 Fig).

**Fig 3:**
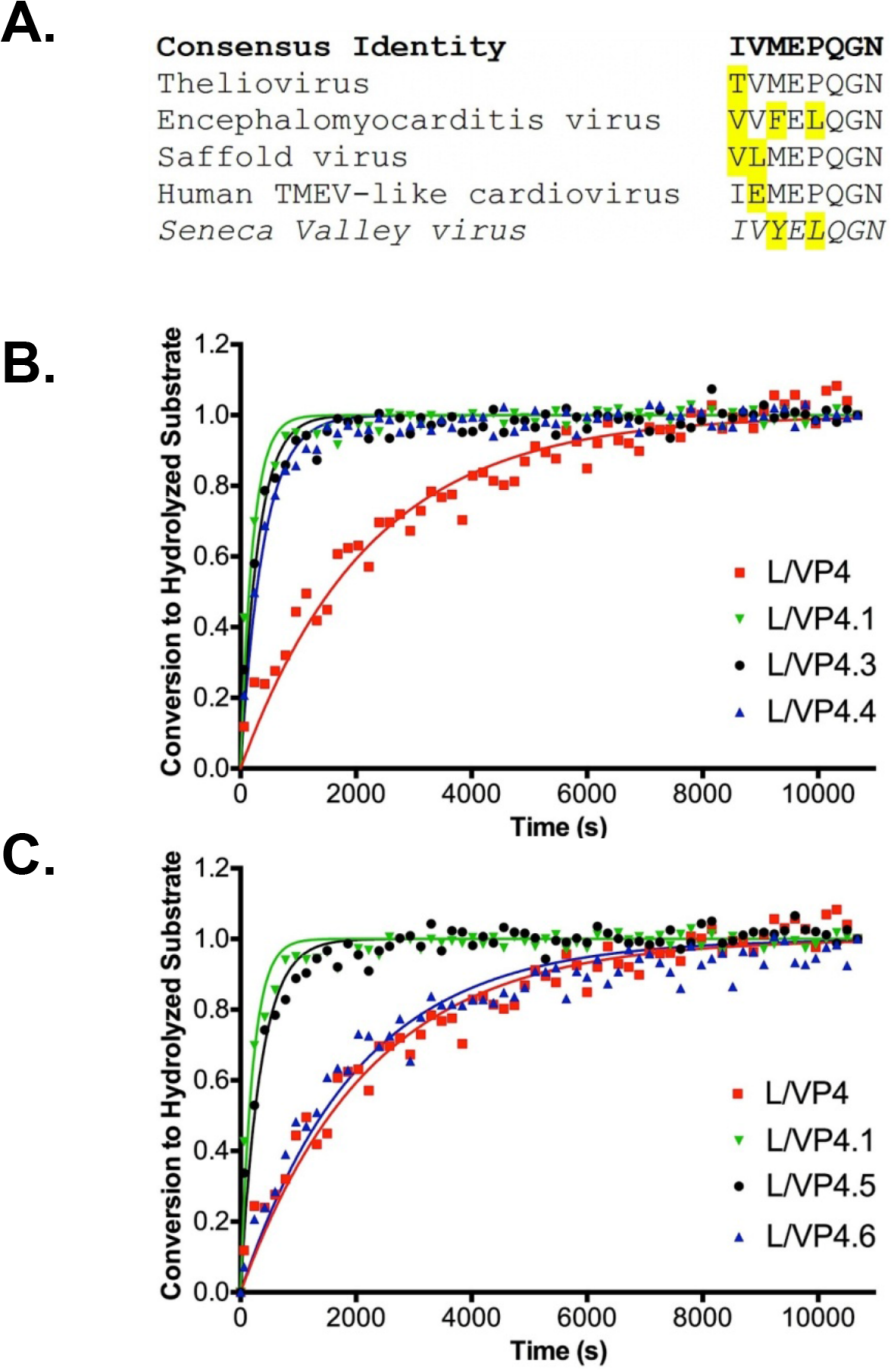
Optimization of the L/VP4 Substrate A. Alignment of SVV L/VP4 cleavage site (shown in italics) with L/VP4 junction sequences of closely related cardioviruses. The amino acids in each sequence that diverge from the consensus sequence (shown in bold) are highlighted. **B.** Conversion of modified L/VP4.1 FRET substrates by purified SVV-001 3C^pro^ over time. Data points represent the average of three replicates at each time point. The endogenous L/VP4 substrate and optimized L/VP4.1 substrate are shown for reference. L/VP4.3 and L/VP4.4 are P4 Y→F substitution and P4 Y→M substitution substrates, respectively. Lines of the same color correspond to curve fits from GraphPad. Error bars on data points were removed for figure clarity. **C.** Conversion of truncated L/VP4.1 FRET substrates by purified SVV-001 3C^pro^ over time. Data points represent the average of three replicates at each time point. The endogenous L/VP4 substrate and optimized L/VP4.1 substrate are shown for comparison. L/VP4.5 and L/VP4.6 are P6 and P5/P6 truncations of L/VP4.1, respectively. Lines of the same color correspond to curve fits from GraphPad. Error bars on data points were removed for figure clarity.

Although the P2’ Pro substitution (L/VP4.1) significantly increases *k*_cat_/*K*_M_, the P2’ Asn is universally conserved in the VP4 proteins of cardioviruses. Amino acids in the non-prime P1-6 sites of the L/VP4 sequence are more divergent (Fig 3A). Thus, the non-prime L/VP4.1 amino acids may be evolutionarily optimized for rapid cleavage under the constraint of a suboptimal P2’ Asn. To determine substrate requirements in these positions, we substituted amino acids found in related cardioviruses in the P2 and P4 sites to create L/VP4.2, (P2 L→P substitution), L/VP.3 (P4 Y→F substitution), and L/VP4.4 (P4 Y→M substitution) (Fig 3B). We also used the L/VP4.1 substrate to determine substrate length constraints for the protease by sequentially removing P6-P3 amino acids from the non-prime side and testing all truncated substrates for cleavage (L/VP4.5-.8; Fig 3C). The FRET ratios from the experiment were used to measure conversion and calculate *k*_cat_/*K*_M_ (Table 1). The substitution made to create L/VP4.2 was detrimental, as this construct was not efficiently cleaved in 3 hours. The L/VP4.3 and L/VP4.4 FP substrates did undergo cleavage more efficiently than the endogenous L/VP4 FP substrate, but 30% and 40% less efficiently than the L/VP4.1 Pro variant, respectively. Removal of the P6 Ile residue in L/VP4.5 decreased the turnover efficiency of cleavage from L/VP4.1 by almost 2-fold but remained a better substrate than the endogenous L/VP4. Removal of both P6 and P5 amino acids (L/VP4.6) decreased the *k*_cat_/*K*_M_ to values similar to the endogenous L/VP4 sequence. Further truncation of the substrate abolished detectable cleavage by SVV-001 3C^pro^ (Table 1).

**Fig 4:**
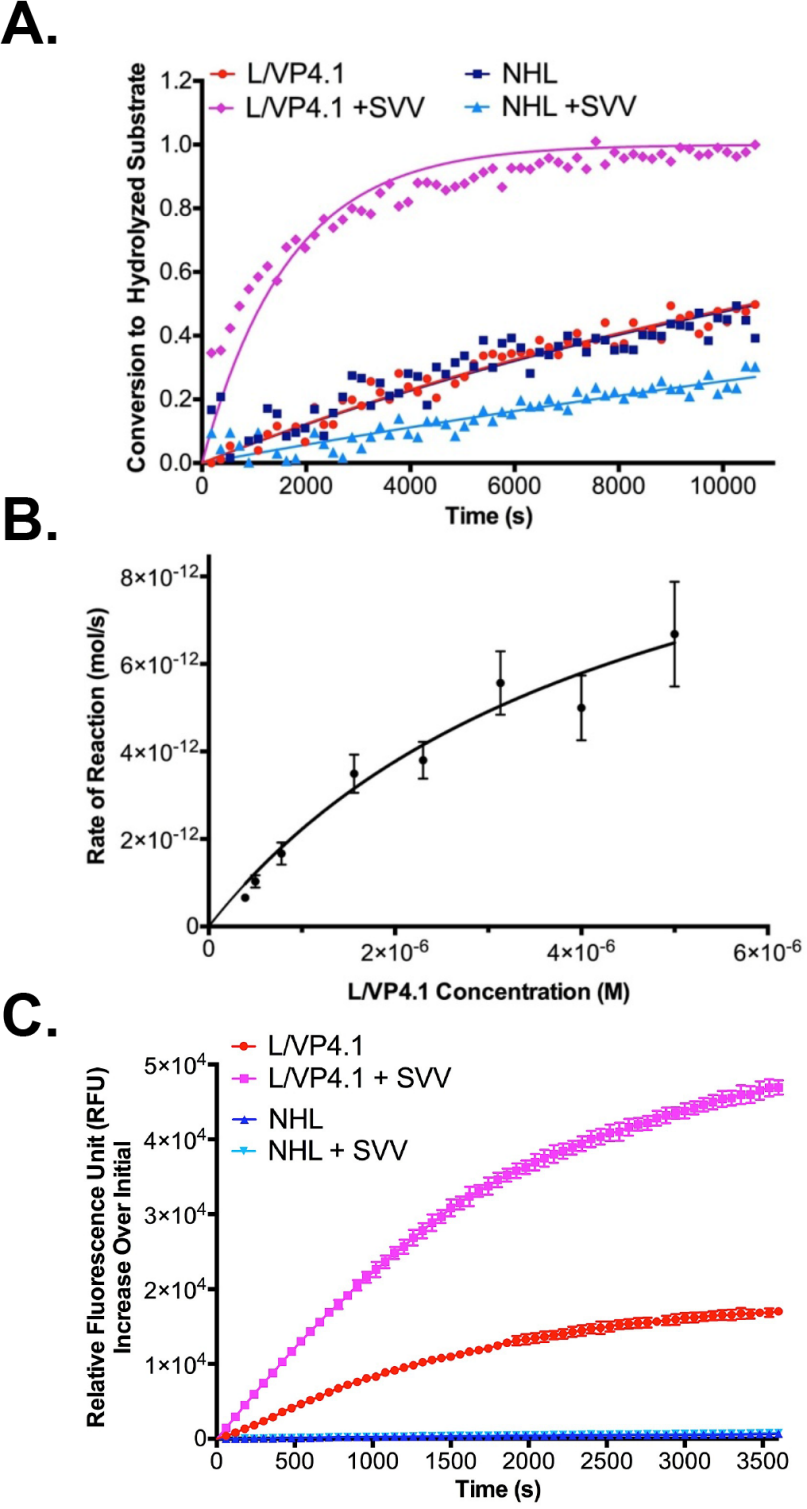
SVV-001 3C^pro^ substrates are cleaved in the context of a cellular infection. **A.** Conversion of FRET substrates by SVV-001 3C^pro^ produced by a cellular SVV infection of permissive SCLC line, NCI-H446. Data points represent the average of three replicates at each time point. The L/VP4.1 FP substrate incubated with uninfected and infected cells, using similar incubations of NHL FP substrate as a negative control. Lines of the same color correspond to curve fits from GraphPad. Error bars on data points were removed for figure clarity. **B.** Initial reaction rates of L/VP4.1 peptide cleavage by recombinant SVV-001 3C^pro^. Data points represent the initial rate of reaction at each concentration of L/VP4.1 peptide calculated from three replicate experiments. Data points were fit to a Michaelis-Menten nonlinear regression from GraphPad and the kinetic constants determined by the curve fit were reported. Standard deviation values of the kinetics constants were calculated by the GraphPad software and propagated through second order rate constant calculations. **C.** Proteolysis of CPQ2/5-FAM peptides by native SVV-001 3C^pro^ in a cellular assay with NCI-H446. Data points represent the average relative fluorescence units (RFUs) increase of three replicates at each time point relative to fluorescence at time zero. The L/VP4.1 FQ peptide was incubated with uninfected and infected cells, using similar incubations of NHL FQ peptide as a negative control. Lines of the same color correspond to connecting line between points.

Determination of an optimized amino acid sequence and length for efficient recognition and proteolysis described above was performed using the recombinant SVV-001 3C^pro^. To verify that the lead substrate would be actively cleaved by the native 3C^pro^, the L/VP4.1 FP substrate was evaluated as a substrate for proteolysis catalyzed by SVV-001 3C^pro^ produced during an active SVV-001 infection, in which 3C^pro^ released during a lytic viral infection is the sole source of the 3C^pro^. Substrates were incubated for 3 hours at 37°C with the SCLC cell line, NCI-H446, after an 8 hour infection with SVV-001 (Fig 4A). Eight hours after infection, cells infected with SVV undergo cell lysis releasing both new SVV virions as well as 3C^pro^ [7]. Loss of FRET, indictative of cleavage, was only observed when the L/VP4.1 FP substrate was incubated with SVV-001 infected H446 cells. Incubations of either FP substrate with uninfected cells, or infected cells with the NHL FP substrate, did not show a loss of FRET over 3 hours. With the second order rate constant previously calculated for the L/VP4.1 FP substrate, we determined the concentration of 3C^pro^ during the SVV infection to be 30.4 ± 4.9 nM using the equation described in Boulware et al [25]. Since loss of FRET was observed exclusively in the presence of infected cells, we conclude that proteolysis of the L/VP4.1 FP substrate is catalyzed selectively by SVV-001 3C^pro^ and not cellular proteases expressed by H446 cells.

We considered the possibility that the FRET fusion peptide substrate for SVV-001 3C^pro^ could influence peptide cleavage kinetics. Thus, a second approach to determine the efficiency of cleavage of L/VP4.1 by SVV-001 3C^pro^ employed a 5-FAM/CPQ2 fluorophore/quencher pair incorporated into the peptide (Fig 4B). In this case, SVV-001 3C^pro^ catalyzed peptide cleavage will result in an increase in fluorescence as 5-FAM is released through proteolysis. The peptide substrate, synthesized between a 5-FAM/CPQ2 fluorophore/quencher pair, was incubated at concentrations ranging from 7 μM to 500 nM with 50 nM recombinant 3C protease for 1 hour at 37°C. The NHL substrate was also synthesized as an FQ peptide and used as a negative control. From the initial rates of reaction, we determined the second order rate constant (*k*_cat_/*K*_M_) for the L/VP4.1 peptide to be 5.42 ± 1.17 × 10^5^ M^-1^s^-1^. The difference in specificity constants may be attributable to the different contexts in which the substrate sequence is presented. In the L/VP4.1 FP substrate, two large proteins flank the relatively short substrate, which could be limiting access of the SVV-001 3C^pro^ despite the presence of a flexible linker. Conversely, the fluorophore and quencher in the L/VP4.1 FQ peptide is much smaller in size and therefore may better reflect the context of the L/VP4.1 substrate in the native SVV-001 polyprotein.

The L/VP4.1 FQ peptide was also tested for proteolysis by the native 3C^pro^ produced during a cellular SVV infection. Using the NHL FQ peptide as a negative control, the substrates were incubated at 37°C for 3 hours with NCI-H446 cells, following an 8 hour infection with SVV-001 (Fig 4C). Similar to the cellular assay with FP substrates, cells infected with SVV are undergoing cell lysis 8 hrs post infection, releasing both new SVV virions as well as 3C^pro^ [7]. A significant increase in fluorescence, indicative of cleavage of the FQ peptide, was only observed in the incubation of the L/VP4.1 FQ peptide with SVV-001 infected cells. The NHL FQ peptide was not cleaved in incubations with either uninfected or infected H446 cells. The L/VP4.1 FQ peptide incubated with uninfected cells also did not show an appreciable increase in fluorescence. The initial rates of reaction were determined to be 0.168 ± 0.0025 pmol/sec and 0.446 ± 0.0056 pmol/sec for the L/VP4.1 FQ peptide incubated with uninfected and SVV infected cells, respectively. As with the cellular FRET assay, we conclude that the L/VP4.1 FQ peptide is selectively cleaved by SVV-001 3C^pro^.

**Fig 5:**
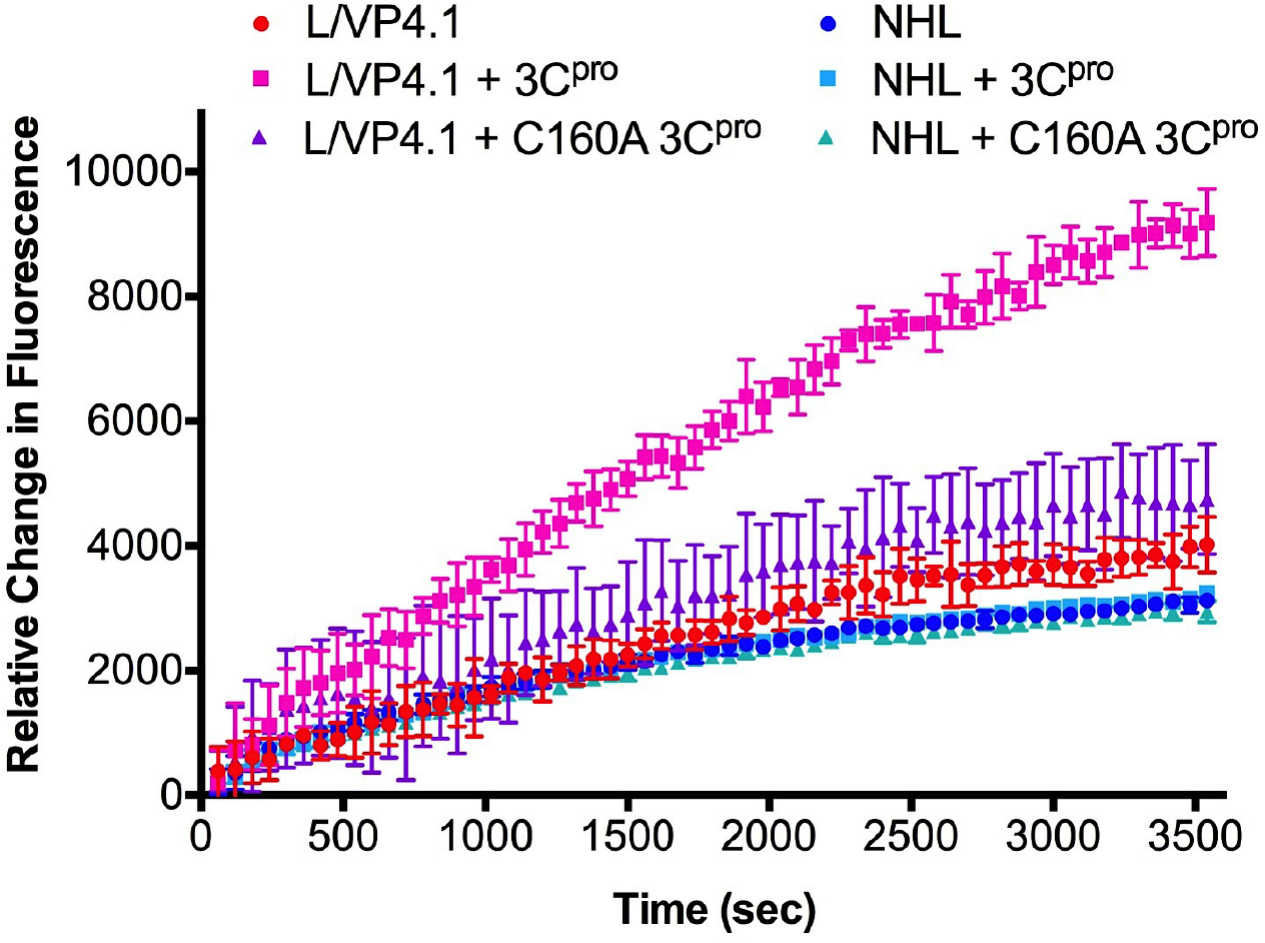
Stability of L/VP4.1 fluorescently quenched peptide in human plasma. Data points represent the average relative fluorescence units (RFUs) increase of four replicates at each time point relative to fluorescence at time zero for L/VP4.1 FQ peptide or NHL FQ peptide incubated with human plasma. The peptides were also incubated with human plasma supplemented with SVV 3C^pro^ or SVV C160A 3C^pro^ as positive and negative controls, respectively. Uncertainty is expressed by standard deviation.

A peptide prodrug based on the L/VP4.1 substrate would most likely be administered intravenously, therefore it was important to determine the stability of the peptide in human plasma (Fig 5). Both the L/VP4.1 and NHL FQ peptides were incubated at a final concentration of 20 μM with a 50% plasma/water solution for 1 hour at 37°C. There was no significant change in fluorescence for either the NHL or L/VP4.1 FQ peptide relative to plasma alone. Therefore, we conclude that the L/VP4.1 FQ peptide is stable in human plasma.

## Discussion

Proteases participate in a large number of essential functions, including but not limited to DNA transcription, cell proliferation, signaling cascades, microenvironment remodeling, and cell death [28]. Their roles in these processes depend on the intrinsic activity of the protease and its ability to selectively cleave its substrate(s). Because of their innate specificities, many cellular proteases have been used to selectively activate peptide prodrugs. The regulated release of the active cytotoxic agent is designed to reduce toxic side effects of the parent drug while increasing its specificity for the targeted cells [8]. Although there have been success stories in the peptide prodrug field, many prodrugs have failed preclinical tests because of premature activation at sites other than the tumor. Redundancy in substrate specificity and non-canonical substrates can lead to non-specific prodrug activation, increasing possible side effects and decreasing efficacy [29].

Anticipating these issues, we focused on designing an optimized substrate that was efficiently and specifically cleaved by SVV-001 3C^pro^. The use of the FP substrates and FQ peptides allowed us to follow kinetic assays in real-time with a purified recombinant form of the 3C^pro^ as well as the native SVV-001 3C^pro^ produced during a cellular infection. The FRET protein substrates had the added advantage of being easily modified by cloning techniques, allowing us to test and identify multiple optimized substrates that were specifically cleaved by the protease [25, 30]. Cellular experiments with the L/VP4.1 FP substrate and FQ peptide ultimately determined the specificity of the SVV-001 3C^pro^ for the L/VP4.1 substrate produced during an SVV-001 infection and allowed us to estimate the total 3C^pro^ concentration in extracellular space.

We have shown that the recombinant fusion SVV-001 3C^pro^ rapidly cleaved two of the ten proposed 3C^pro^ substrates. Efficient substrate recognition and hydrolysis by many proteases is not solely based on the primary amino acid sequence; secondary and in some cases, tertiary structures also play a role in regulating proteolytic cleavage. For a majority of picornaviral polyproteins, protein precursors begin to undergo folding before cleavage between the individual proteins even occurs [31, 32]. In the context of an isolated protease substrate, important structural features of the polyprotein are lost, which could substantially alter or even abolish substrate hydrolysis. However, a ribosome skipping event following the 2A peptide positions both the L/VP4 and 2B/2C sites at the amino terminal ends of nascent peptides, making them easily accessible to SVV-001 3C^pro^, and potentially reducing complex secondary and tertiary structure [20, 33]. Their intrinsic lack of rigid structure may explain why only these two substrates were efficiently cleaved by SVV-001 3C^pro^ in the context of the FP substrates.

Viral fusion proteins, intermediates in the proteolytic cascade, may also play a role in efficiency of substrate recognition and cleavage. Fusion proteins such as 3CD may be responsible for some of the cleavage events in the SVV-001 polypeptide [34, 35]. We have not ruled out the possibility that some of the proposed 3C^pro^ substrates in SVV-001 are better substrates for fusion proteins like 3CD than for mature isolated 3C^pro^. A final caveat is that the purified SVV-001 3C^pro^ used in these studies is a fusion protein with MBP. Although it is possible that the presence of MBP may affect the cleavage specificity or kinetics of the protease, this is likely to be a minor effect, as other viral proteases have been purified as fusion proteins and substituted for their normal counterparts in a similar fashion showing no significant change in specificity or activity [36]. The use of this construct was required for these investigations, as the fusion protein, but not isolated SVV-001 3C^pro^, could be maintained in solution after proteolytic removal of MBP with TEV protease [22, 24]. Recognition of the L/VP4.1 FP substrate and FQ peptide by the native SVV-001 3C^pro^ during a lytic viral infection confirms the feasibility of using the recombinant SVV-001 3C^pro^ in initial substrate optimization.

While we were successful at developing a highly optimized protease substrate, it is important to note that the rational approach used for optimization did not interrogate all potential substrates of SVV 3C^pro^. Approaches for protease substrate identification have been developed that are capable of screening a large sequence space for substrates with efficient turnover [26, 37]. L/VP4.1 has the potential to be optimized further using one or a combination of these approaches to test for extended substrates or to test for all possible combinations of P1-P4 residues, potentially leading to L/VP4.1 variants with improved properties.

An ideal substrate for a peptide prodrug is one that is not cleaved by any other protease found in the human body. Unlike the substrates usually conjugated in other peptide prodrugs, the L/VP4.1 substrate described here is a viral polyprotein sequence cleaved by a viral protease. Because neither the protease nor substrate is found in uninfected human cells, this approach substantially lowers the chance of non-specific recognition and activation by cellular proteases. To confirm our hypothesis, we performed an *in silico* search of the MEROPS database which contains cleavage data on over 3,000 proteases including human and human pathogens [38]. No human proteases were predicted to cleave L/VP4.1. A single hit for Southampton virus, a calicivirus that causes acute gastroenteritis was also noted [39]. Therefore, any peptide prodrug which utilizes a viral protease and substrate pair, such as an L/VP4.1 based prodrug, is likely to have superior selectivity relative to prodrugs activated by endogenous cellular proteases.

A prodrug based on the L/VP4.1 substrate introduces a novel form of VDEPT based on a wild-type virus. This strategy combines multiple advantages of the antibody-directed enzyme prodrug therapy (ADEPT) and VDEPT concepts while overcoming a few of the limitations. No genetic engineering of the virus is needed because the wild type virus already contains active protease and specifically homes to the tumor site. With this approach, the virus plays a more important role than solely as a transport or targeting vector for the enzyme gene; this strategy incorporates the intrinsic ability of the virus to lyse cancer cells in combination with the peptide-prodrug to increase the therapeutic effect on the tumor. The existence of both an intracellular and extracellular pool of SVV 3C^pro^ during a cellular infection allows for increased flexibility when designing the peptide prodrug [40].

SVV-001 has been shown to selectively infect cancers with neuroendocrine features, including SCLC and pediatric brain tumors, both of which are in desperate need for new effective therapies [2]. Although SVV-001 has been shown to be effective *in vitro* and in patient derived xenografts of variant SCLC, high viral titers are needed to eradicate tumors *in vivo* [5]. Promising results from preclinical mouse models, and initial experience in patients with advanced cancer, has prompted clinical evaluation of SVV-001 efficacy in both SCLC and pediatric neuroendocrine cancer patients [3, 4, 6, 15]. Our proposed approach to improving the efficacy of SVV-001 is to combine virotherapy with a peptide prodrug of a potent cytotoxic agent, activated by SVV-001 3C^pro^. A prodrug of this kind is expected to be highly selective because of the high specificity of the virus as well as the uniqueness of the SVV-001 3C^pro^ and its substrates. Such a VDEPT strategy may also allow for a “bystander effect” where uninfected tumor cells could be killed by the active cytotoxic agent released by neighboring infected cells [41, 42]. An added benefit of this novel combination is the increased effect on tumor cells before neutralizing antibodies are produced against the virus and render it inactive. A novel combination of this kind may prove to be a highly effective therapy for multiple SVV-permissive cancer types. Finally, these data represent a proof of concept for other oncolytic viruses with highly active and sequence specific proteases.

This study characterized the substrate requirements of the SVV 3C^pro^ and identify an optimized substrate with high turnover efficiency for incorporation into a protease activated peptide prodrug. The substrate studies performed here have identified a lead substrate efficiently cleaved by the SVV-001 3C^pro^, which may be used as the basis for a peptide prodrug amenable to combination with SVV virotherapy as a novel form of VDEPT.

## Supporting Information

S1 Fig SDS-PAGE gel of overexpression and purification of recombinant fusion SVV-001 3C^pro^ and catalytically dead mutant SVV-001 C160A 3C^pro^. Lane 1- Molecular weight protein marker. Lane 2- Insoluble protein after cell lysis (1:10 dilution). Lane 3- Soluble protein after cell lysis. Lane 4- Pool eluted fractions from HisPur cobalt resin purification of 3C protease. Lane 5- Amicon Ultra-15 50K MWCO flow through. Lane 6- Concentrated elution fractions after Amicon Ultra-15. The band corresponding to the recombinant fusion SVV-001 3C^pro^ and SVV-001 C160A 3C^pro^ is labeled.
(TIF)

S2 Fig Conversion of Substrates after Incubation with Increased Concentration of SVV-001 3C^pro^. All substrates except the L/VP4 and 2B/2C were incubated with 1 μM purified SVV-001 3C^pro^ for 3 hours at 30°C. The decrease in FRET was measured in real time using a fluorimeter and data was converted to display fraction of substrate converted to hydrolyzed substrate over time. Data points represent the average of three replicates at each time point. The HRV 3C^pro^ substrate was used as a positive control. 2C/3A, 3B/3C, and 3C/3D are endogenous substrates that showed cleavage. All other substrates were not cleaved in the presence of increased SVV-001 3C^pro^ (data not shown).
(TIF)

S3 Fig Conversion of Substrates after Incubation with SVV-001 3C^pro^ compared to SVV-001 C160A 3C^pro^. Substrates L/VP4 and L/VP4.1 were incubated with 250 nM purified SVV-001 3C^pro^ (labeled WT) or SVV-001 C160A 3C^pro^ (labeled C160A) for 3 hours at 30°C. The decrease in FRET was measured in real time using a fluorimeter and data was converted to display fraction of substrate converted to hydrolyzed substrate over time. Changes in FRET from substrates incubated with C160A 3C^pro^ were divided by total change in FRET by substrates incubated with WT 3C^pro^ indicating complete hydrolysis. Data points represent the average of three replicates at each time point.
(TIF)

S4 Fig Comparison of SVV 3C^pro^- L/VP4.1 Substrate Specificity to Selected Picornavirus Protease-Substrate Pairs. **A.** Substrates L/VP4.1 (labeled SVV), HRV, and Coxsackievirus (CAV) 2C/3A substrate (labeled CAV) with amino acid sequence MEALFQ↓GP were incubated with 250 nM SVV 3C^pro^ (black data points), HRV 3C^pro^ (blue data points), or CAV 3C^pro^ (red data points) for 3 hours at 30°C. CAV 3C^pro^ was cloned, overexpressed, and purified using the same methods described for SVV 3C^pro^ in the Methods section. The decrease in FRET was measured in real time using a fluorimeter and data was converted to display fraction of substrate converted to hydrolyzed substrate over time. Data points represent the average of three replicates at each time point. **B.** Heat map depicting relative rates of cleavage for protease-substrate pairs compared to the native protease-substrate pair.
(TIF)

S5 Fig Comparison of Conversion of Substrate containing Q↓SP cleavage site to L/VP4 and L/VP4.1 Substrates. Substrate L/VP4.9 with amino acid sequence IVYELQ↓SP was cloned between the FRET pair, CyPET and YPET using primer numbers 21–22 (S1 Table), induced for overexpression, and purified using methods described for FRET substrates in the main text. Substrates L/VP4, L/VP4.1, and L/VP4.9 were incubated with 250 nM purified SVV-001 3C^pro^ for 3 hours at 30°C. The decrease in FRET was measured in real time using a fluorimeter and data was converted to display fraction of substrate converted to hydrolyzed substrate over time. Data points represent the average of three replicates at each time point.
(TIF)

S1 Table Oligonucleotide list for primers used for the construction of FRET fusion proteins and recombinant SVV-001 3C^pro^. Numbers correspond to the primer identity in the main text. Primer name describes the product the primer will eventually create. Oligonucleotides shown from 5’ to 3’.
(PDF)
