# Correction: Seneca Valley Virus 3C^pro^ Substrate Optimization Yields Efficient Substrates for use in Peptide-Prodrug Therapy

**DOI:** 10.1371/journal.pone.0136480

**Published:** 2015-08-19

**Authors:** Linde A. Miles, W. Nathaniel Brennen, Charles M. Rudin, John T. Poirier

In the Materials and Methods section, there is an error in the equation in the section titled “In Vitro Cleavage Assay.” Please view the complete, correction equation:

$$Conversion=1-\exp(-\frac{k_{cat}}{K_{M}}[E]∙t)$$
