# Supplementary figures and images for: Seneca Valley Virus 3Cpro Substrate Optimization Yields Efficient Substrates for Use in Peptide-Prodrug Therapy
Source: PLoS One. 2015 Jun 12;10(6):e0129103. doi: 10.1371/journal.pone.0129103 (PMC4466507; doi:10.1371/journal.pone.0129103)

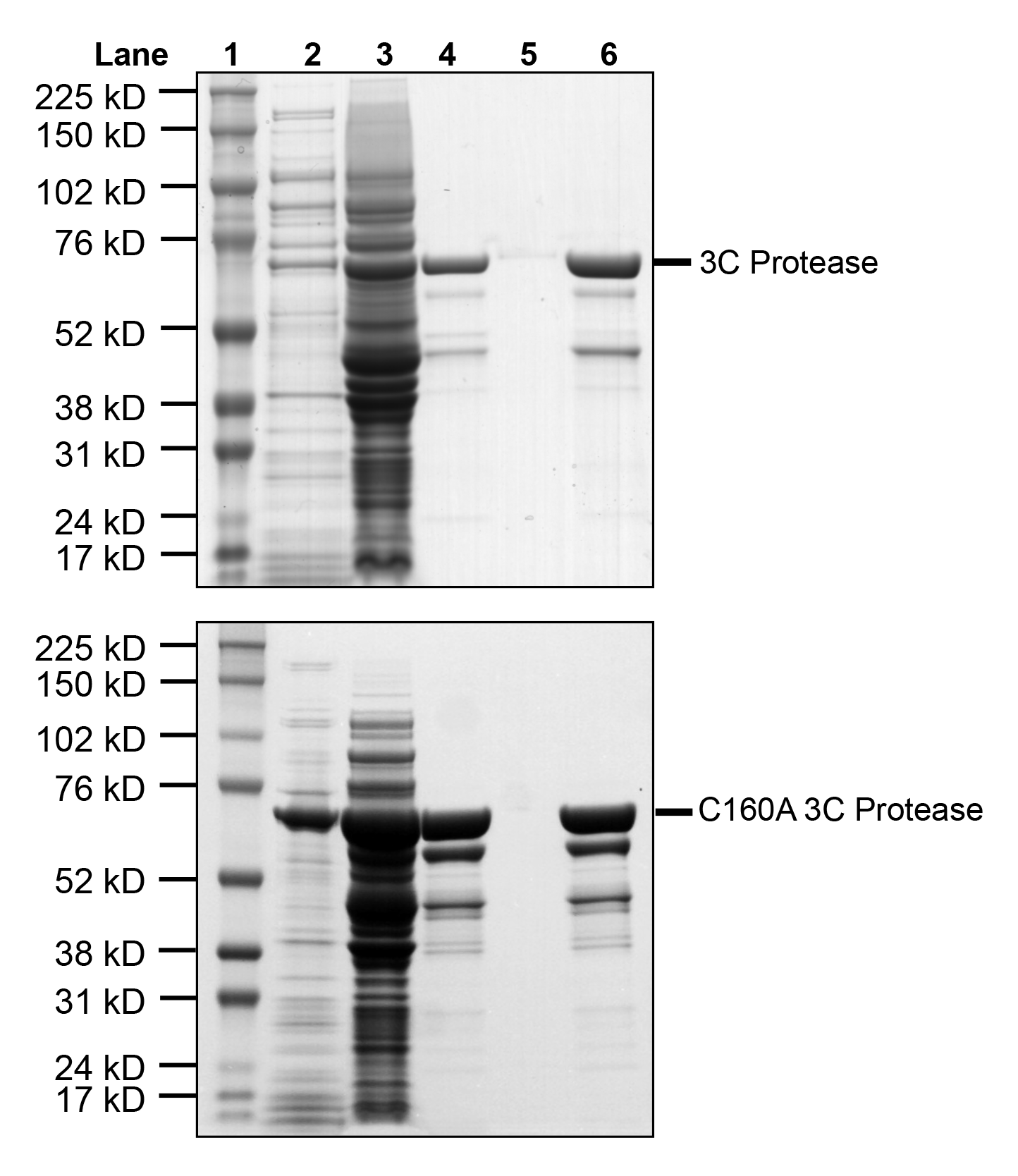

Supplement: S1 Fig — Lane 1- Molecular weight protein marker. Lane 2- Insoluble protein after cell lysis (1:10 dilution). Lane 3- Soluble protein after cell lysis. Lane 4- Pool eluted fractions from HisPur cobalt resin purification of 3C protease. Lane 5- Amicon Ultra-15 50K MWCO flow through. Lane 6- Concentrated elution fractions after Amicon Ultra-15. The band corresponding to the recombinant fusion SVV-001 3Cpro and SVV-001 C160A 3Cpro is labeled. (TIF) [file pone.0129103.s001.tif]

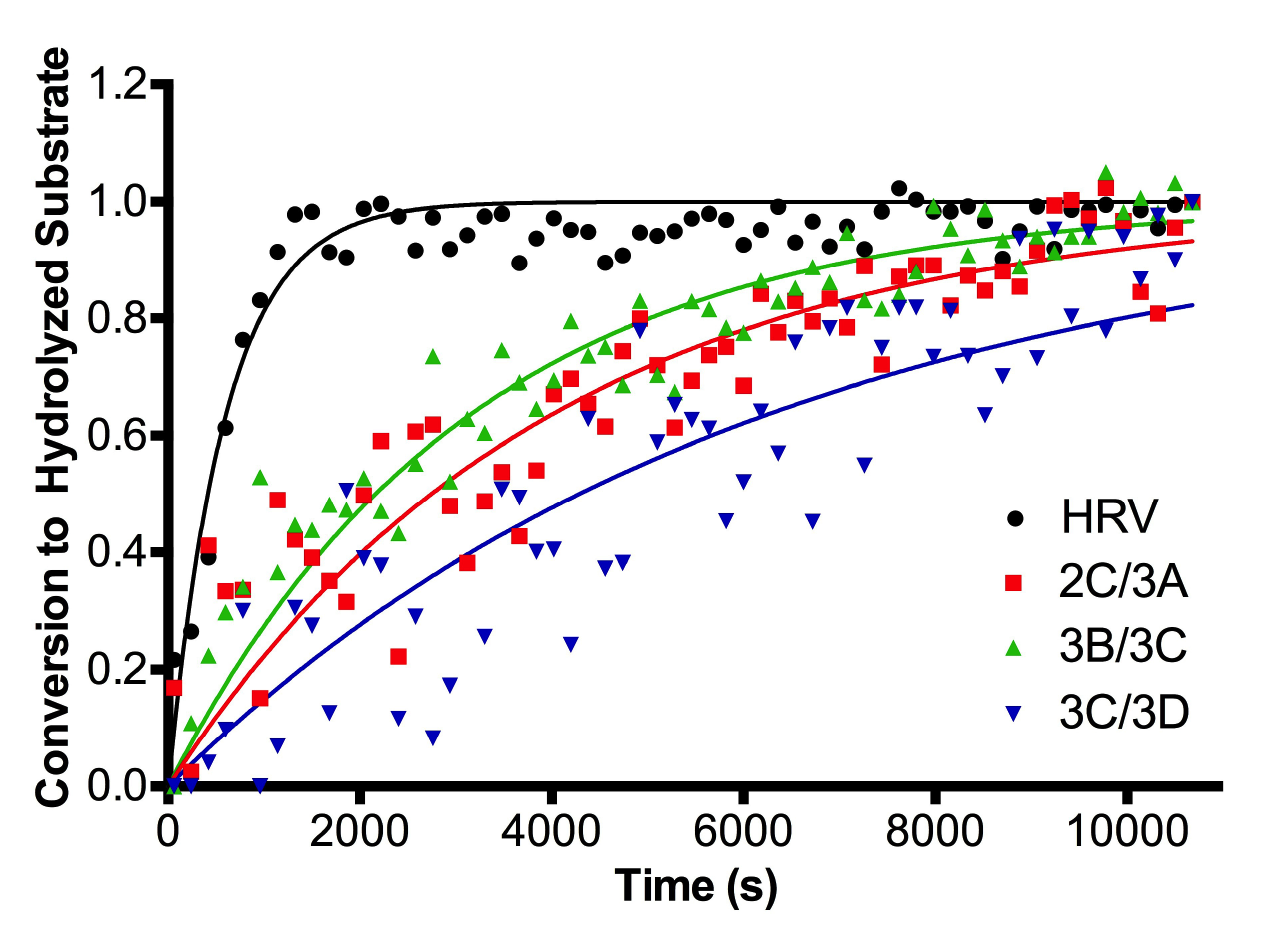

Supplement: S2 Fig — All substrates except the L/VP4 and 2B/2C were incubated with 1 μM purified SVV-001 3Cpro for 3 hours at 30°C. The decrease in FRET was measured in real time using a fluorimeter and data was converted to display fraction of substrate converted to hydrolyzed substrate over time. Data points represent the average of three replicates at each time point. The HRV 3Cpro substrate was used as a positive control. 2C/3A, 3B/3C, and 3C/3D are endogenous substrates that showed cleavage. All other substrates were not cleaved in the presence of increased SVV-001 3Cpro (data not shown). (TIF) [file pone.0129103.s002.tif]

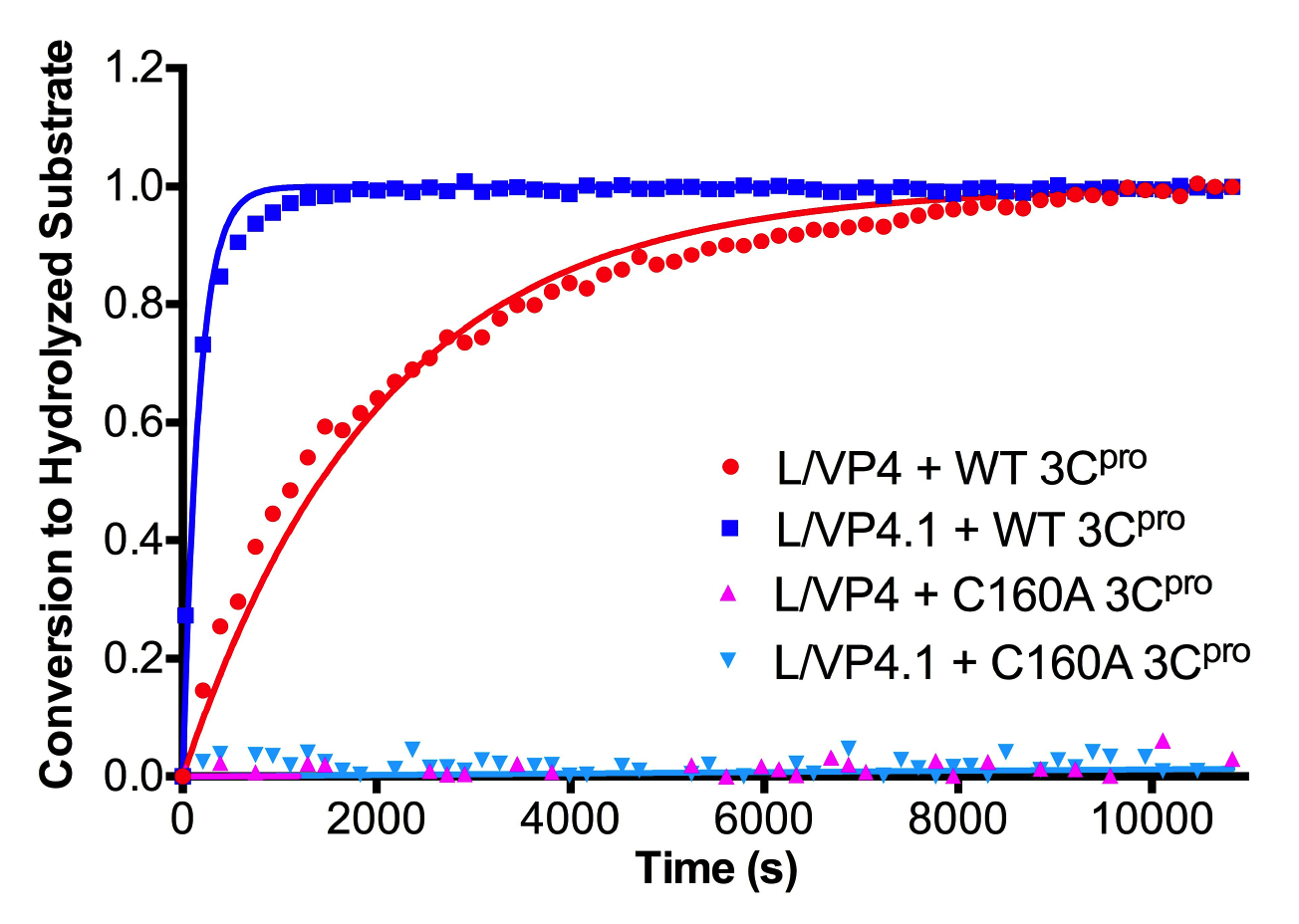

Supplement: S3 Fig — Substrates L/VP4 and L/VP4.1 were incubated with 250 nM purified SVV-001 3Cpro (labeled WT) or SVV-001 C160A 3Cpro (labeled C160A) for 3 hours at 30°C. The decrease in FRET was measured in real time using a fluorimeter and data was converted to display fraction of substrate converted to hydrolyzed substrate over time. Changes in FRET from substrates incubated with C160A 3Cpro were divided by total change in FRET by substrates incubated with WT 3Cpro indicating complete hydrolysis. Data points represent the average of three replicates at each time point. (TIF) [file pone.0129103.s003.tif]

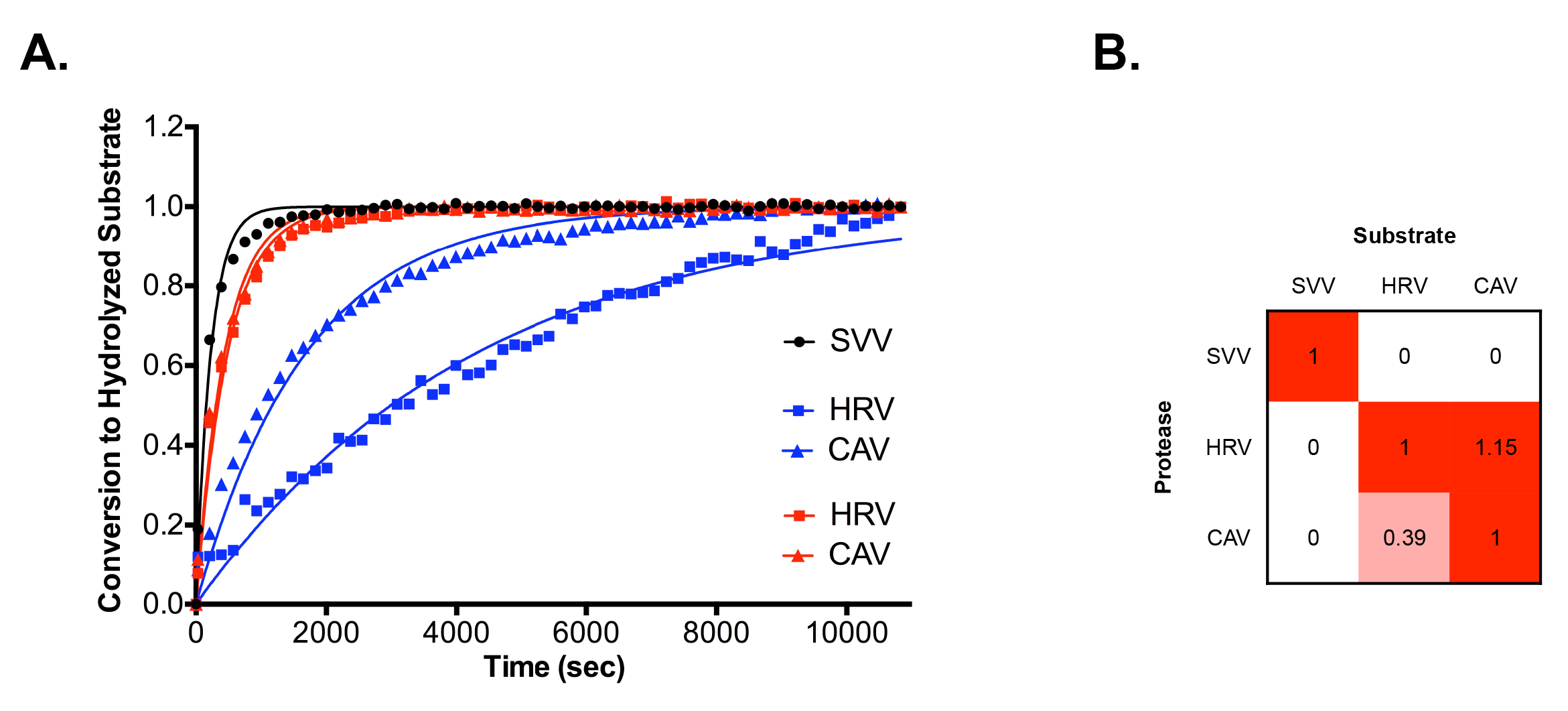

Supplement: S4 Fig — A. Substrates L/VP4.1 (labeled SVV), HRV, and Coxsackievirus (CAV) 2C/3A substrate (labeled CAV) with amino acid sequence MEALFQ↓GP were incubated with 250 nM SVV 3Cpro (black data points), HRV 3Cpro (blue data points), or CAV 3Cpro (red data points) for 3 hours at 30°C. CAV 3Cpro was cloned, overexpressed, and purified using the same methods described for SVV 3Cpro in the Methods section. The decrease in FRET was measured in real time using a fluorimeter and data was converted to display fraction of substrate converted to hydrolyzed substrate over time. Data points represent the average of three replicates at each time point. B. Heat map depicting relative rates of cleavage for protease-substrate pairs compared to the native protease-substrate pair. (TIF) [file pone.0129103.s004.tif]

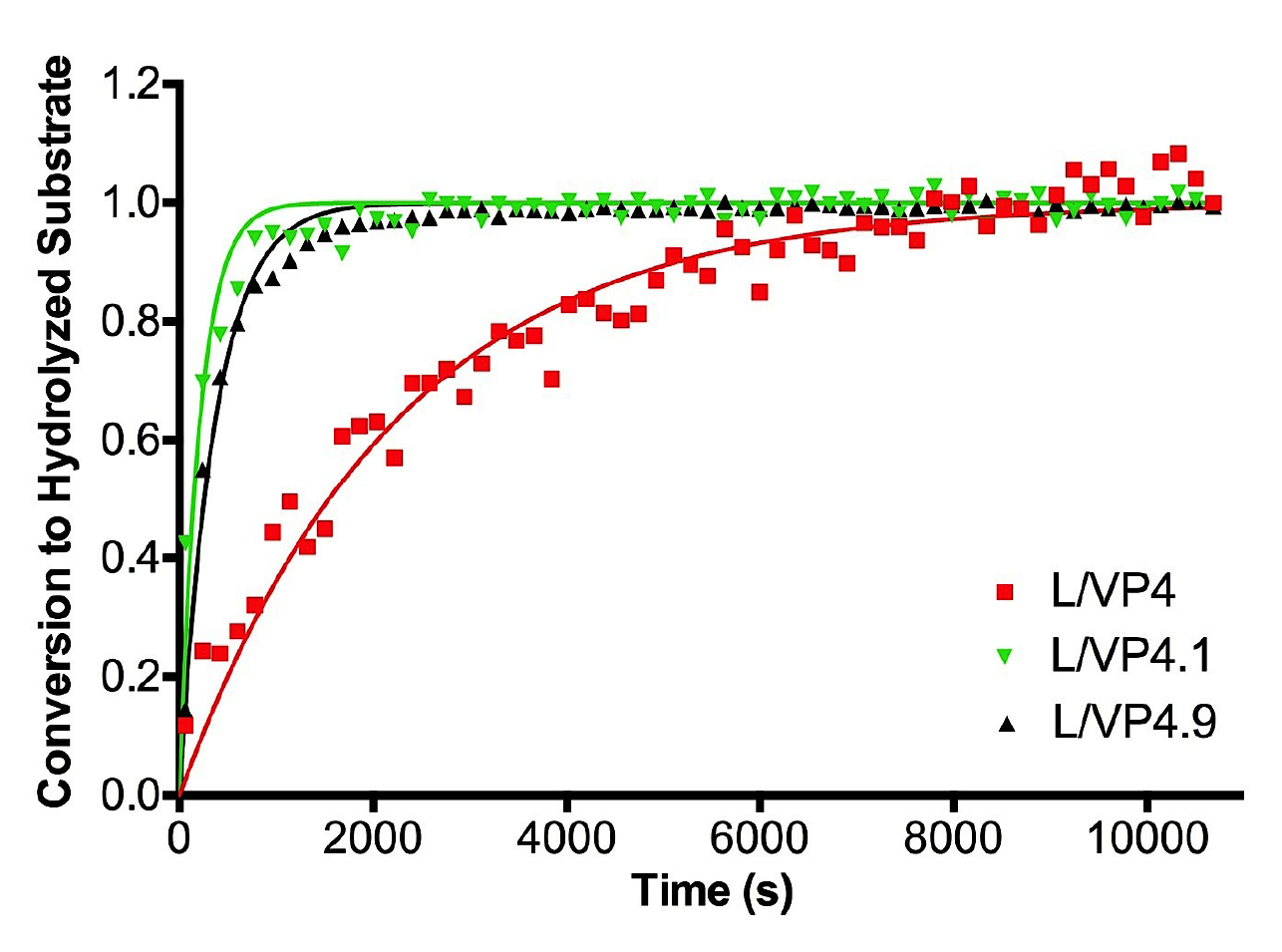

Supplement: S5 Fig — Substrate L/VP4.9 with amino acid sequence IVYELQ↓SP was cloned between the FRET pair, CyPET and YPET using primer numbers 21–22 (S1 Table), induced for overexpression, and purified using methods described for FRET substrates in the main text. Substrates L/VP4, L/VP4.1, and L/VP4.9 were incubated with 250 nM purified SVV-001 3Cpro for 3 hours at 30°C. The decrease in FRET was measured in real time using a fluorimeter and data was converted to display fraction of substrate converted to hydrolyzed substrate over time. Data points represent the average of three replicates at each time point. (TIF) [file pone.0129103.s005.tif]
