# Supplementary material for: Seneca Valley Virus 3Cpro Substrate Optimization Yields Efficient Substrates for Use in Peptide-Prodrug Therapy
Source: PLoS One. 2015 Jun 12;10(6):e0129103. doi: 10.1371/journal.pone.0129103 (PMC4466507; doi:10.1371/journal.pone.0129103)
Supplement: S1 Table — Numbers correspond to the primer identity in the main text. Primer name describes the product the primer will eventually create. Oligonucleotides shown from 5’ to 3’. (PDF) [file pone.0129103.s006.pdf]

Name	Primer Sequence
1-HRV	5’-GGGGTACCCTGGAAGTTCTGTTCCAGGGTCCGGGTGGTGGTAGCGGTGG-3’
2-L/VP4	5’-GGGGTACCATCGTTTACGAACTGCAGGGTAACGGTGGTGGTAGCGGTGG-3’
3-VP4/VP2	5’-GGGGTACCCCGCTGGGTTACCTGAAAGACCACGGTGGTGGTAGCGGTGG-3’
4-VP2/VP3	5’-GGGGTACCGGAACCGACGAAGAACAGGGTCCGGGTGGTGGTAGCGGTGG-3’
5-VP3/VP1	5’-GGGGTACCCCGTCTTACGTTTTCCACTCTACCGGTGGTGGTAGCGGTGG-3’
6-VP1/2A	5’-GGGGTACCCAGAAAATGCTGATGCAGTCTGGTGGTGGTGGTAGCGGTGG-3’
7-2B/2C	5’-GGGGTACCAAACTGTTCAAAATGCAGGGTCCGGGTGGTGGTAGCGGTGG-3’
8-2C/3A	5’-GGGGTACCCAGACCCTGGTTCTGCAGTCTCCGGGTGGTGGTAGCGGTGG-3’
9-3A/3B	5’-GGGGTACCAAAGCTCCGCGTTCTGAAAACGCTGGTGGTGGTAGCGGTGG-3’
10-3B/3C	5-GGGGTACCTCTCTGATGGAAATGCAGCAGCCGGGTGGTGGTAGCGGTGG-3’
11-3C/3D	5’-GGGGTACCCCGCTGGCTACCATGCAGGGTCTGGGTGGTGGTAGCGGTGG-3’
12-L/VP4.1	5’-GGGGTACCATCGTTTACGAACTGCAGGGTCCGGGTGGTGGTAGCGGTGG-3’
13-2B/2C.1	5’-GGGGTACCAAACTGTTCAAAATGCAGGGTAACGGTGGTGGTAGCGGTGG-3’
14-L/VP4.2	5-GGGGTACCATCGTTTACGAACCGCAGGGTCCGGGTGGTGGTAGCGGTGG-3’
15-L/VP4.3	5’-GGGGTACCATCGTTTTCGAACTGCAGGGTCCGGGTGGTGGTAGCGGTGG-3’
16-L/VP4.4	5’-GGGGTACCATCGTTATGGAACTGCAGGGTCCGGGTGGTGGTAGCGGTGG-3’
17-L/VP4.5	5’-GGGGTACCGTTTACGAACTGCAGGGTCCGGGTGGTGGTAGCGGTGG-3’
18-L/VP4.6	5’-GGGGTACCTACGAACTGCAGGGTCCGGGTGGTGGTAGCGGTGG-3’
19-L/VP4.7	5’-GGGGTACCGAACTGCAGGGTCCGGGTGGTGGTAGCGGTGG-3’
20-L/VP4.8	5’-GGGGTACCCTGCAGGGTCCGGGTGGTGGTAGCGGTGG-3’
21-L/VP4.9	5’-GGGGTACCATCGTTTACGAACTGCAGAGCCCGGGTGGTGGTAGCGGTGG-3’
22-pB33CGSY R	5’-ACATGCATGCGGCCACCTT-3’
23-3CProtease F	5’-GGGGACAAGTTTGTACAAAAAAGCAGGCTTCGAAAACCTGTACTTCCAGGA TTATAAAGATGAACAGCCCAACGTGGACAT-3’
24-3CProtease R	5’-GGGGACCACTTTGTACAAGAAAGCTGGGTTTATTGCATTGTGGCCAAAGG-3’
25-C160A 3CProtease F	5’-TACAAAGGATGGGCCGGCTCGGCCCTG-3’
26-C160A 3CProtease R	5’-GATGTTTCCTACCCGGCCGAGCCGGGA-3’
